# Clinical Aspects in Subacute Thyroiditis: A Real-Life Study on 226 Cases in Greece Amid the COVID-19 Pandemic

**DOI:** 10.3390/jcm12227171

**Published:** 2023-11-18

**Authors:** Nikolaos Angelopoulos, Dimitrios P. Askitis, Ioannis Androulakis, Nicolas Valvis, Rodis Paparodis, Valentina Petkova, Anastasios Boniakos, Dimitra Zianni, Ilias Perogamvros, Konstantinos Toulis, Sarantis Livadas, Ioannis Iakovou

**Affiliations:** 1 Academic Department of Nuclear Medicine, School of Medicine, AHEPA University Hospital, 54636 Thessaloniki, Greece; iakovou@otenet.gr; 2 Athens Medical Centre, 65403 Athens, Greece; dimitrios.askitis@gmail.com (D.P.A.); iandroulakis@gmail.com (I.A.); valvisnikos@yahoo.com (N.V.); valiapetg@gmail.com (V.P.); anbonendo74@gmail.com (A.B.); dimzianni@hotmail.com (D.Z.); sarntis@gmail.com (S.L.); 3 Center for Diabetes and Endocrine Research, College of Medicine and Life Sciences, University of Toledo, Toledo, OH 43606, USA; rodis@paparodis.gr; 4 Division of Diabetes, Endocrinology and Gastroenterology, School of Medical Sciences, University of Manchester, Manchester M13 9PL, UK; ilias.perogamvros@manchester.ac.uk; 5 Diabetes Unit, Division of Endocrinology, 1st Department of Internal Medicine, AHEPA University Hospital, 54636 Thessaloniki, Greece; touliskos@gmail.com

**Keywords:** subacute thyroiditis (SAT), SARS-CoV-2 complications, natural history, hypothyroidism, recurrences

## Abstract

Purpose: This study aimed to evaluate various therapeutic approaches, identify potential predictive factors for the recurrence and development of hypothyroidism, and examine specific clinical and laboratory characteristics of patients with subacute thyroiditis (SAT) due to SARS-CoV-2 infection. Methods: We retrospectively analyzed the medical records of 226 patients with confirmed SAT diagnosed from January 2020 to November 2022. Results: The mean age was 48.01 ± 0.75 years, and the F/M ratio was 2.3/1. At the end of the follow-up period, 69 patients (32.1%) had developed hypothyroidism. Treatment duration was significantly shorter with nonsteroidal anti-inflammatory drugs (NSAIDs) (17.40 ± 2.56 days), while time-to-symptom relief was shorter with glucocorticoids (CGs). Recurrence was observed only in those treated with corticosteroid preparations (14.1%). C-reactive protein levels at treatment discontinuation were higher in patients who experienced SAT recurrence, while the coexistence of Hashimoto’s thyroiditis was a significant predictive factor for the development of hypothyroidism. The TSH value at the time of treatment withdrawal >4.12 μIU/mL showed optimal sensitivity and specificity for the prediction of permanent hypothyroidism. Regarding COVID-19, 34 patients (15%) experienced related SAT, with similar clinical manifestations of the disease but a higher BMI and shorter time-to-symptom relief. Conclusion: In conclusion, GCs administration alleviated acute symptoms earlier during the onset of SAT, whereas NSAIDs had a shorter treatment duration, and both regimens could not prevent the development of delayed hypothyroidism. The clinical characteristics of SAT due to COVID-19 infections were similar to those of typical SAT disease.

## 1. Introduction

Subacute thyroiditis (SAT) (also called De Quervain’s thyroiditis or granulomatous thyroiditis) is a self-limited, inflammatory thyroid disorder of viral origin commonly associated with neck pain and various systemic symptoms [1]. SAT exhibits a seasonal distribution that coincides with the peak incidences of viral infection of the upper respiratory system (coxsackie virus A and B, influenza, echovirus infections), and most recently, SARS-CoV-2 infections (COVID-19). The highest incidence of SAT occurs in middle-aged women, and females account for 75–80% of all SAT patients [2]. The most characteristic laboratory finding is a high erythrocyte sedimentation rate (ESR), sometimes reaching even three-digit values. The C-reactive protein (CRP) is elevated in many cases, although it is a less common typical marker. Anti-thyroid antibodies are usually normal. The characteristic ultrasound (US) pattern of SAT includes hypoechoic and heterogeneous areas with blurred margins, poorly vascularized on color Doppler [2,3,4,5], and has largely eliminated the use of iodine or technetium thyroid scintigraphy in terms of final diagnosis. Susceptibility to SAT has long been linked to specific human leukocyte antigen (HLA) types, with HLA-B35 previously identified as a correlate. Recent research has expanded this understanding, revealing associations between SAT occurrence and the presence of additional HLA alleles, notably HLA-B*18:01, -DRB1*01, and -C*04:01 [3].

Although the natural course of the disease is believed to be often self-limiting, patients frequently suffer from severe symptoms that persist even for months and rarely resolve only after treatment with glucocorticoids (GCs), nonsteroidal anti-inflammatory drugs (NSAIDs), or even in combination. The selection of medication is generally based on clinical experience, and the majority of studies in the literature are retrospective cohort studies without any specific basis for the treatment options preferred [4,5].

The recurrence of thyroiditis is frequently encountered and may be attributed to the different preferred anti-inflammatory therapies, duration, and dose of treatment. Furthermore, age may have an impact on conflicting findings reported by several investigators [6]. Nevertheless, permanent hypothyroidism constitutes the main long-term consequence of SAT, presenting in approximately 6–15% of patients [7,8]. On clinical grounds, there is a paucity of data regarding potential predictive factors for subsequent hypothyroidism development in affected patients. The purpose of this retrospective study was to estimate the rate of recurrence and the development of hypothyroidism in a large number of patients with SAT and investigate the presence of possible risk factors. We further investigated the occurrence of specific clinical and laboratory characteristics to present practical information on the COVID-19 infection-related SAT as a new entity among cases of De Quervain’s thyroiditis.

## 2. Materials and Methods

### 2.1. Patients

A retrospective multicenter review of 22,517 medical charts was conducted in eleven high-volume private Endocrinology, Diabetes, and Metabolism outpatient clinics. Charts considered initially were patients with a referred diagnosis of SAT from January 2020 to November 2022. The diagnosis was based on the following criteria (as recently proposed by Stasiak and Lewiński in 2021). Clinical symptoms of painful, tender goiter are often accompanied by signs and symptoms resembling an upper respiratory infection with fever and malaise and an elevation of ESR levels with either depressed radioactive iodine uptake (RAIU) or ultrasonographic findings compatible with a diagnosis of subacute thyroiditis [2]. We excluded 12 cases with a doubtable diagnosis or insufficient follow-up (<3 months).

Demographical (age, gender, Body mass index), hormonal [Thyroid-stimulating hormone (TSH), Free thyroxine (FT4), Thyroid peroxidase antibodies (anti-TPO), Anti-thyroglobulin antibodies (anti-TG)], serological (ESR, CRP), and treatment parameters (type of therapy, duration of therapy, initial dose of treatment, time to treat, time to symptoms relief) were also reviewed as potential risk factors for disease recurrence and the development of hypothyroidism. A relapse of clinical symptoms accompanied by an increase in ESR levels after treatment discontinuation was considered a disease recurrence. Detections of thyroid nodules >5 mm in patients with previously normal thyroid ultrasound were assumed to be new cases of nodular disease. For methodological purposes, the glucocorticoid potency of the various GC drugs used in this study was calculated as the equivalent dose of methylprednisolone.

TSH and FT4 were measured by a sandwich electrochemiluminescence immunoassay (ECLIA) on Cobas 8000 (Roche, Basel, Switzerland) and Abbott Architect (Architect; Abbott, Chicago, IL, USA). For Abbott Architect, the normal reference range was TSH 0.4–4.2 μIU/mL and FT4 0.8–1.5 ng/dL. For Roche Cobas 6000, the normal reference range was TSH 0.27–4.20 μIU/mL and FT4 0.93–1.7 ng/dL. Thyroglobulin (Tg) was measured by Abbot Architect and IMMULITE 2000® analyzer (Siemens, Los Angeles, CA, USA) and Abbot Architect (Abbott Laboratories, Abbott Park, IL, USA), with a normal range of 1.6–60 ng/mL. Thyroid autoantibodies (anti-TPO and anti-TG) were measured with chemiluminescent microparticle immunoassays (CMIA).

This study’s procedures were approved by the Institutional Review Board (IRB protocol number 10446) of AHEPA Hospital, Thessaloniki, Greece. The protocol employed in this study was conducted following the tenets of the Declaration of Helsinki. Due to the retrospective nature of the study, neither patient approval nor informed consent were required.

### 2.2. Statistical Analysis

Data analysis was performed using SPSS for Windows v24 statistical software (Statistical Package for Social Science, SPSS Inc., Chicago, IL, USA). Fisher’s exact or Chi-square tests were used to compare categorical variables. Student’s t-test and the non-parametric Mann–Whitney test were used to compare continuous variables, which were presented as mean and standard deviation unless otherwise addressed. When comparing more than two samples, one-way ANOVA was used for normally distributed values and the Kruskal–Wallis test was used for data that were not normally distributed. The factors that were tested to affect the dependent variable (hypothyroidism or recurrence) were included in a logistic regression analysis model to determine the independent factor. For diagnostic purposes, the cutoff value was determined using the Receiver Operating Characteristic (ROC) curve. The value corresponding to the maximum summation of sensitivity and specificity was taken as the cutoff value. A *p*-value of <0.05 was considered significant.

## 3. Results

The data originated from the medical records of 226 (158 women and 68 men) Greek patients of Caucasian origin with an SAT age range of 18–86 years. The baseline demographic and laboratory characteristics of patients are illustrated in Table 1. The mean age was 48.01 ± 0.75 (SEM) years, F/M (ratio: 2.3/1). At baseline, overt hypothyroidism was present in 11 patients (4.86%), while a known history of Hashimoto’s disease was present in 40 (four of them with nodules), and thyroid nodular disease was present in 30 (defined as the presence of one or more thyroid nodules ≥5 mm in diameter in ultrasound examination). One patient was treated for hyperthyroidism, and the rest, 144, had no known thyroidal disease.

At the end of the follow-up period (16.77 ± 0.8 months, mean ± SEM), overt clinical hypothyroidism was diagnosed in 44 patients, and it was thus recommended that they receive levothyroxine (L-T4) replacement therapy.

In 31 patients who exhibited increased TSH levels (TSH > 10 mIU/L) in two consecutive measurements, 2–4 months after the onset of SAT, low doses of thyroxine were prescribed. According to their medical records, the thyroxin dose was adjusted accordingly to thyroid function tests after 4–6 weeks to justify the permanent need for hormonal replacement. Treatment discontinuation was successfully applied in 6 out of the 31 patients, while the TSH value rose again in 25 of them. Finally, 69 previously euthyroid subjects (32.1%) were designated as having permanent hypothyroidism.

In 99 of 144 patients with an initially normal thyroid, no signs of thyroidal deterioration were observed. Regarding treatment allocation, 156 (69%) patients were treated with GCs: 109 were treated with methylprednisolone (MEP), 7 with dexamethasone (DXA), 40 with prednisolone (PSL), 42 with the NSAID ibuprofen (IBU), 15 with a combination of PSL and IBU, and 13 received no therapy. Final hypothyroidism in previously euthyroid patients was more frequent in those with Hashimoto thyroiditis (*p* = 0.026, coefficient of the variable b = 0.88, odds ratio 2.41, 95% confidence interval 1.2–4.83).

**Figure 1:**
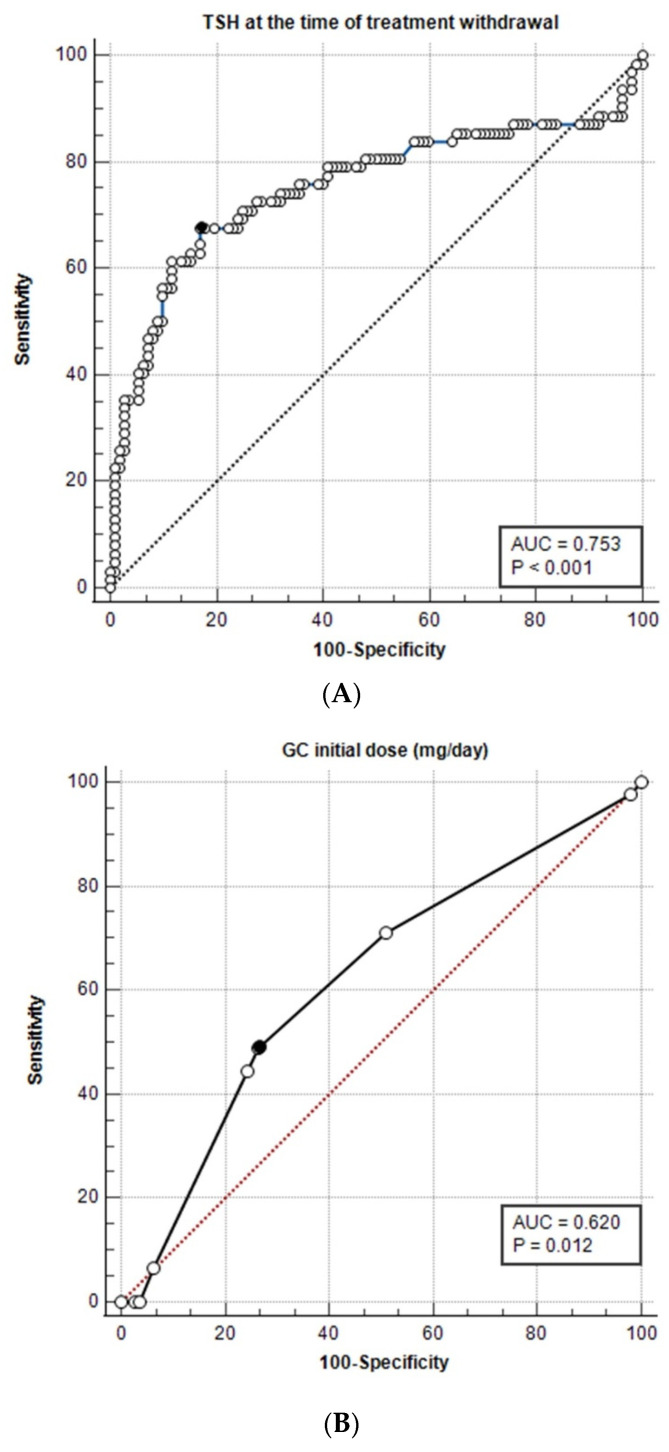

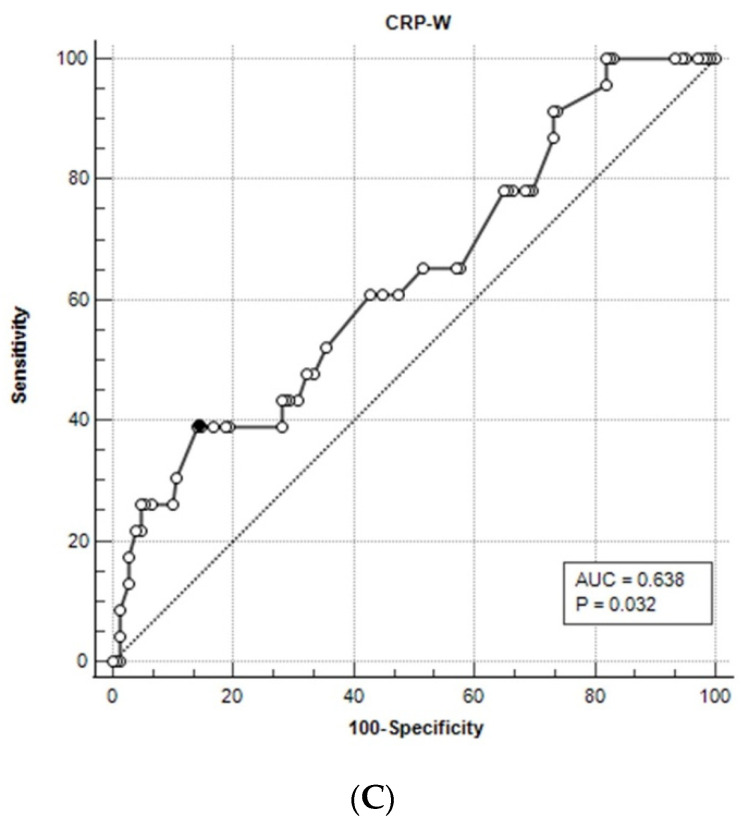
(**A**) Youden index 0.508 is illustrated with a black circle, associated criterion > 4.12 μIU/mL, sensitivity 67.74%, and specificity 83.04%. (**B**) GC, glucocorticoid dose equivalent to methylprednisolone. Youden index 0.225 is illustrated with a black circle, associated criterion ≤ 20 mg/day, sensitivity 48.89%, and specificity 73.64%. (**C**) CRP-W, CRP values at the time of therapy withdrawal; Youden index 0.250 is illustrated with a black circle; associated criterion 2.6 mg/L; sensitivity 39%; specificity 86%.

A step-wise logistic regression analysis was performed to evaluate the factors associated with the development of hypothyroidism. Demographical (age, gender, BMI), serological (ESR, CRP), hormonal (TSH, FT4, Tg, anti-TPO, anti-TG), before treatment initiation and at the time of therapy discontinuation. Additionally, treatment parameters (type of therapy, duration of therapy, initial dose, time to treat, and time to symptom relief) were also analyzed. After excluding non-significant variables, the model of TSH at the time of withdrawal, time to treatment, and duration of therapy was significant (Chi-square = 40.51, *p* < 0.001). The ROC curve was used to illustrate and evaluate the prognostic value of TSH levels at the time of therapy withdrawal on the development of hypothyroidism (Figure 1A). Results indicated that the cutoff value of TSH >4.12 μIU/mL (Youden’s index 0.508, 95% Confidence interval 0.368 to 0.629) showed an optimal sensitivity of 67.74% and a specificity of 83.04%.

To assess the risk of hypothyroidism among the subgroup of GC-treated patients, ROC curve analysis based on the initial dose of GC (equivalent to methylprednisolone) showed a negative predictive value [coefficient b = −0.06, Odds Ratio 0.94, 95% confidence interval (CI) 0.9–0.99], with an area under curve (AUC) of 0.620 for hypothyroidism vs. euthyroidism (*p* = 0.012; Figure 1B). According to Youden’s index, the best cutoff value for the GC level was ≤20 mg/day, with a sensitivity of 48.89% and a specificity of 73.64%.

Regarding treatment protocols, significant differences were detected for treatment duration (shorter with IBU, *p* < 0.001, Kruskal–Wallis test), time-to-symptom relief (shorter with MEP, *p* < 0.05, Kruskal–Wallis test), and the initial equivalent dose of cortisone (lower in combined treatment with cortisone and ibuprofen, *p* < 0.001, one way ANOVA).

A recurrence was observed in 28 patients (14.1%), and 26 of them were treated with cortisone preparations. A stepwise multiple regression analysis for the prediction of SAT recurrence was performed. The model included all serological indices (TSH, FT4, anti-tpo, anti-Tg, Tg, TKE, and CRP) before and after treatment withdrawal. Except for CRP levels at the time of treatment withdrawal (CRP-W, Coefficient: 0.095, Std. Error: 0.038, t: 2.511, *p*: 0.02), no other predictive factors were found in terms of SAT recurrence rate. Logistic regression analysis was performed to examine the influence of CRP-W on the prediction of SAT recurrence and showed a significant model (Chi-square = 6.699, *p* < 0.01). ROC analysis showed a significant positive coefficient of CRP-W, b = 0.263, *p* = 0.007, Odds Ratio 1.301, 95% CI 1.074–1.576. According to Youden’s index (0.2504), the best cutoff value for the CRP-W level was 2.6 mg/L, and the sensitivity and specificity were 39% and 86%, respectively. The optimal criterion was CRP-W >6 mg/L, with a sensitivity of 17.39% and a specificity of 97.32% (Figure 1C).

Thirty-four patients (15%) had COVID-19-related SAT without any specific clinical features. Two females were hospitalized (4 and 8 days, respectively) before the initiation of SAT symptoms with mild symptoms of pneumonia, while the rest were outpatients’ cases. In the 34 cases that we reviewed, the mean age of patients was 47.5 ± 10.4 years, with a greater female preponderance (74.1%). The mean number of days between the start of the COVID-19 illness and the appearance of SAT symptoms was 15.2 ± 8.1. Four patients were confirmed to have ongoing COVID-19, whereas the infection had resolved in 30 patients before the onset of SAT symptoms. Fever and neck pain were the common presenting complaints in all patients. Eighty-five percent of patients reported some type of hyperthyroid symptoms. In all patients, laboratory analysis confirmed low TSH and increased FT4 values, and ultrasound findings were suggestive of SAT. Twenty patients were unvaccinated (60%), seven were not fully vaccinated (20%), and seven had completed vaccination (three doses of the Pfizer–BioNTech COVID-19 vaccine). Compared to the non-COVID group, BMI (Kgr/m^2^) was higher (27.58 ± 4.05 vs. 25.69 ± 5.44, mean ± SD, respectively, *p* = 0.026), and a shorter time-to-symptom relief was observed (3.56 ± 1.94 vs. 5.46 ± 6.93 days, respectively, *p* = 0.044), as illustrated in Table 2.

## 4. Discussion

The main findings of our study were the significantly higher recurrence rate of SAT in patients who were treated with GCs and a significantly higher rate of hypothyroidism, mainly in those with underlying Hashimoto thyroiditis.

### 4.1. Recurrence

#### 4.1.1. General Characteristics

Recurrences of SAT may occur soon after the initial therapy, but they also happen many years after the first incidence. The rate of SAT recurrence is rather high, despite a proper diagnosis and treatment, and varies between studies from a few to over 20% of cases [9,10].

Our data showed a 14% incidence of recurrences with no particular clinical features in recurrent patients as compared with those without recurrences, according to the recently published meta-analysis by Zhang J [6]. Several studies have reported an age difference for recurrence [5,11,12,13,14] and a lower number of recurrences in males [5,9,11,12,13,15,16]. However, pooled data analysis [15] revealed no significant differences in both age (age mean deference = −0.46, 95% CI: −2.55–1.63) and sex (relative risk = 0.69, 95% CI: 0.41–1.15) between the recurrence and the non-recurrence groups, in line with our findings.

In the largest study published (including more than 3000 patients with SAT), the disease did recur after a prolonged latent period (over a year after the first episode), at least in 2% of patients who had previously demonstrated a complete recovery [14]. However, the mean age of the patients in the first episode who subsequently developed a recurrence was younger than the mean age of the total patients with subacute thyroiditis. Our records concern a long follow-up period (over a year); nonetheless, later recurrences, although rare, cannot be excluded.

#### 4.1.2. Comparison of Therapeutic Approaches

In agreement with our results, it is documented that GCs treatment is superior to NSAIDs in terms of quicker effect and faster pain relief [17,18,19]. In line with our results, the findings of the meta-analysis of Zhang J. [6] suggested that the risk of recurrence among the GC-treated group is higher than that in the NSAID-treated group (RR = 1.84, 95% CI: 1.04–3.24). It has been assumed that GCs are usually used to treat severe patients or patients who do not respond to NSAIDs and are thus more likely to experience recurrence [19,20,21].

#### 4.1.3. Dose of Glucocorticoids

The dose of GCs has also been examined and showed that the recurrence rate of high-dose steroids is higher than that of low-dose steroids [16]. In our cohort, a significantly greater number of patients were treated with methylprednisolone in the recurrence group, and the initial dose was higher (approximately 33 mg of equivalent prednisolone), although the findings did not reach statistical significance. Since the current main purpose of SAT treatment is still to relieve symptoms, medication is generally based on clinical experience, and GCs dosing varies among different treatment protocols. The recommended initial dose of prednisolone is usually 40 mg/day for several weeks, which is gradually tapered [22]. However, high-dose steroids may stimulate virus replication and are more likely to cause recurrence [21]. Several trials have demonstrated that low doses of prednisolone (15–20 mg) may reduce the recurrence rate and have an excellent therapeutic effect [23,24]. Unfortunately, the most appropriate dosage of GCs with the lowest recurrence is still unknown. A combination of a low dose of GCs (mean equivalent dose of 14 mg/day) with NSAIDs showed excellent results in our study in a significant percentage of patients 15 (6.6%) without disease recurrence, which may be used as an alternative option in the future.

#### 4.1.4. Treatment Duration

**Figure 2:**
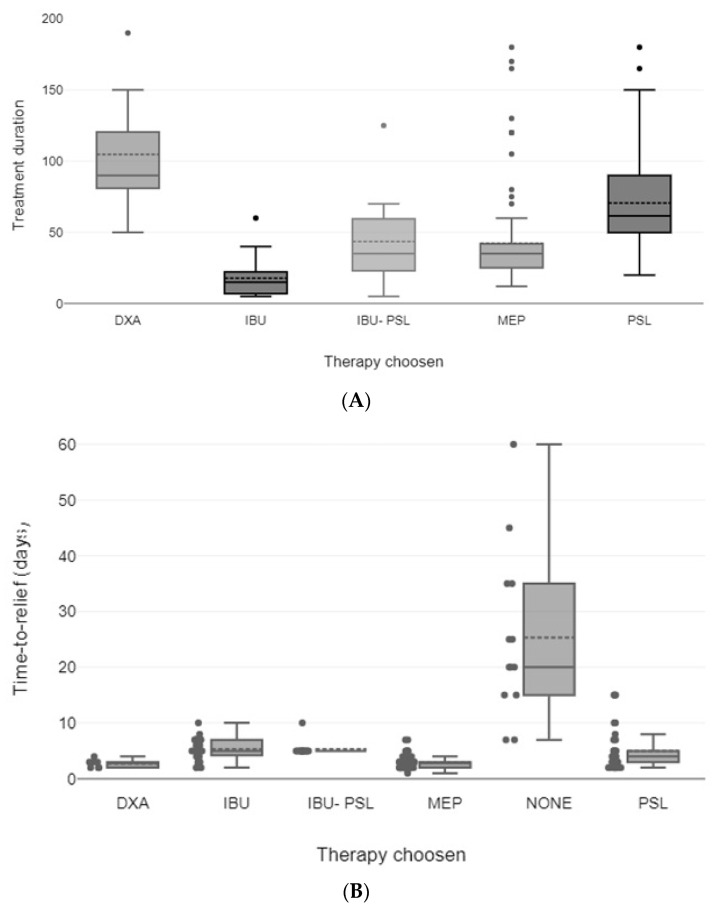
(**A**). Boxplot of the patient’s treatment duration. (**B**). Boxplot of the patient’s time-to-relief. Abbreviations: DXA, dexamethasone; IBU, ibuprofen; PSL, prednisolone; MEP, methylprednisolone; NONE, no therapy. The box indicates the range in which the middle 50% of all data are located. The solid line indicates the median, and the dashed line is the mean value. T-shaped whiskers indicate the last point, which is still 1.5 times the interquartile distance. All values above and below the whisker are outliers. Treatment duration and time to relief are expressed in days.

Although a line of data suggested treatment duration as a potential risk factor for SAT recurrences, we failed to detect such a difference in our patients (Figure 2A). During prednisolone treatment, when tapering from 10 mg/day to 5 mg/day, SAT recurrence is most likely to occur, and extending the duration of treatment by at least six weeks may decrease the recurrence rate [9,12]. In the aforementioned studies, treatment duration was 5 to 6 weeks, and more recurrences were observed when treatment was stopped earlier. However, the mean duration of therapy in our cohort was longer (approximately 41 days), probably explaining the absence of a difference between patients with and without recurrences.

#### 4.1.5. Time to Treat

It has been reported that patients who initiated GCs treatment within 30 days of onset revealed a higher recurrence rate than those who experienced a longer duration of illness before treatment initiation [13]. A possible explanation could be that chronic inflammation of the gland may improve with the natural progression of the disease. No significant difference in time-to-treat was observed in our patients, although it was slightly shorter in the recurrent group. Of note, none of our patients who did not receive anti-inflammatory drugs developed recurrence, although obviously, clinical relief was much longer. This observation raises the question of whether, at least in mild clinical disease, treatment should be avoided.

#### 4.1.6. Time to Clinical Relief

As mentioned before, time to symptom relief is significantly shorter when GC is used compared to NSAIDs and non-treated patients, but no difference was detected in terms of recurrence rate in our cohort (Figure 2B). Again, little is known about the utility of this clinical parameter since its estimation remains mainly empirical.

#### 4.1.7. Laboratory Findings

Recent studies have demonstrated that the severity of inflammation may not predict recurrence in SAT [11,25]. In our study, patients who experienced SAT recurrences appeared to have greater levels of CRP at the time of therapy discontinuation compared to those without a recurrence. It has been previously postulated that too-fast tapering of the GC dose may be one of the reasons for recurrence [12]. A cut-off value of 2.6 mg/L found in our study could serve as a useful clinical criterion when deciding to terminate GC since approximately 90% of recurrences were observed in patients with greater CRP values at the time of therapy withdrawal. In a recently published study, the median TSH level of the patients who had recurrence was 0.3 µIU/mL (range, 0.01–2.38 µIU/mL). Moreover, the optimal TSH cutoff value for the prediction of recurrence was 0.045 µIU/mL, and 83.3% of patients who developed recurrence had TSH values > 0.045 µIU/mL [26]. We did not find a significant difference in any other inflammatory recurrent patients or nonrecurrent patients, as previously reported [27].

### 4.2. Hypothyroidism

Transient hypothyroidism is common in patients with SAT, but permanent hypothyroidism is infrequent, accounting for 6–15% of SAT patients during the follow-up period [7,8]. We found a significantly higher rate of permanent hypothyroidism, arising at 32.1% in patients with initially normal thyroid function. This discrepancy may be attributed in part to the misdiagnosed phenomenon of transient hypothyroidism, which led to earlier therapy and the different cut-off values of thyroid hormones, where clinicians decide to initiate thyroxine supplementation.

In line with the results of our study, previous reports revealed an increased incidence of hypothyroidism after SAT in patients with positive thyroid antibodies up to 72.7% [4,28] while others failed to detect any significant correlation between elevated anti-thyroid peroxidase antibodies and hypothyroidism [27].

Inflammation indices, namely ESR and CRP, have been widely examined as predictive risk factors for hypothyroidism, with heterogeneous findings. CRP levels with a cut-off value of 97.80 mg/L have been reported as a risk factor for hypothyroidism [29], and ESR was significantly associated with persistent subclinical hypothyroidism [30]. On the other hand, no effect of CRP and ESR levels on hypothyroidism has been reported by several authors [7,11,25]. Our study showed that both ESR and CRP had no significant association with hypothyroidism.

In a recently published study, the early maximum TSH value was closely related to the incidence of hypothyroidism at 1 and 2 years after the onset of SAT [25]. The authors have proposed a TSH level cutoff of 7.83 µIU/mL (within 3 months of the onset of SAT) with sensitivity and specificity of 80.0 and 80.6%, respectively. Our research revealed similar results, although follow-up was shorter and the incidence of hypothyroidism was greater in our cohort.

Among GCs treated patients, high doses may interfere with virus replication. In contrast, suboptimal GC dosing may lead to prolonged treatment duration, and ensuing subclinical inflammation may result in permanent thyroid cell lesions. Whether this phenomenon is predominantly dose- or time-dependent (or even both), and in what magnitude, remains questionable.

According to our results, long-term inflammation resulted in the continuous destruction of thyroid cells, and the occurrence of hypothyroidism [7], as observed in a small retrospective study, took a longer time and treatment related to final hypothyroidism. A very interesting finding in our study was the negative impact of time and treatment period on the development of hypothyroidism. We assume that immediate initiation therapy for SAT patients with higher initial doses, although preferable to rapidly eliminate inflammation, may lead to an increase in hypothyroidism in the long term. Furthermore, extending treatment duration may have negative effects on thyroid tissue and increase the risk of hypothyroidism. Nevertheless, the pre-existence of autoimmune thyroiditis seems to be a strong risk factor for hypothyroidism in our study. Along with the acute destructive effects on thyroid follicular cells, the possible triggering of autoimmune processes might be involved with this observation.

#### Adverse Events

Common side effects due to the use of GCs have been previously reported in SAT patients, namely weight gain, glucose intolerance, hypertension, and irregular menses emergence [26]. Interestingly, severe side effects were absent in our study, while mild adverse effects were reported only in the GCs treated group. One 38-year-old female was led to a thyroidectomy due to four consecutive episodes of painful recurrences within 15 months. One 58-year-old male developed autoimmune hyperthyroidism 35 days after SAT therapy with prednisolone was ended. Thereafter, he was successfully treated with methimazole for 6 months with an absolute remission of hyperthyroidism. The diagnosis of Graves’ disease (GD) was documented by both increased thyroid-stimulating immunoglobulin (TSI) levels and radioactive iodine uptake (RAIU). Since similar reported cases are rare [31,32], the implication of SAT infection in triggering an autoimmune response has been proposed as a potential underlying mechanism. Genetic factors, specifically the presence of HLA alleles associated with GD or SAT, may increase susceptibility to the development of thyroid autoimmunity after SAT [33].

### 4.3. Subacute Thyroiditis Induced by COVID-19 Infection

The term “post-viral thyroiditis” seems more appropriate for post-COVID-19 thyroiditis since literature shows that symptoms of SAT usually occur within days or weeks after infection [34,35]. Regarding the clinical manifestation of the disease, available data conflict, since some studies reported more serious symptoms (intense neck pain and a higher incidence of fever) while we, among others, showed a similar profile to other forms of SAT [36]. It has to be noted that all of our COVID-19 patients were diagnosed on an outpatient basis and were mildly symptomatic (or completely asymptomatic), which may in part explain the absence of a more severe clinical form of thyroiditis.

Of interest, in our study, patients with COVID-19 SAT were treated with a higher initial equivalent dose of cortisone, which probably resulted in a shorter time to symptom relief. Indeed, no one was treated with either a combination of cortisone and NSAIDs (where the cortisone dose is usually lower) or DXA. It seems that, while confronting the uncertainty of a new clinical entity with an absence of guidelines and despite the mild form of the disease, physicians choose to be more aggressive when facing COVID-19 patients. Nevertheless, concerning short-term (recurrences) and long-term (hypothyroidism) consequences, no differences among other forms of SAT were revealed in our cohort by the available published reports [36]. Since the current cumulative amount of statistical evidence is low, a general conclusion about the preferable treatment approach in COVID-19 relates to SAT. The age range was 35–86 years, and typical patients were middle-aged adult (47.53 ± 10.42, mean ± SD) females (ratio 3.9:1), in compliance with other studies [36]. Of note, we detected a slight but significant increase in BMI in patients with COVID-related SAT. A growing body of evidence suggests that patients who are overweight or obese are more likely to develop severe COVID-19 and multiple organ lesions [37]. Several mechanisms of thyroid damage have been proposed in COVID-19 disease, including host immune overreaction, immune deficiency related to infection, destruction of lymphocytes, inhibition of the innate immune response, and direct cellular destruction with apoptosis [38]. Since the exact pathophysiological processes underlying SAT development after COVID-19 disease are still unclear, to what extent our finding is of clinical importance is unknown.

Inflammation markers, namely SER and CRP, are positive in the majority of SAT cases, but we did not detect any difference among COVID-19 and SAT patients. In accordance, current data have failed to document any clear correlation between the clinical presentation of SAT after COVID-19 infection and inflammatory markers, nor was a highly suggestive marker of potential thyroid comorbidities identified in a patient developing coronavirus infection [30,39,40,41,42].

#### Limitations and Areas of Uncertainty

Our study has several limitations due to its retrospective nature. First, the patients received treatment not based on randomization but on their clinical condition and caring physician approach; this might have caused selection bias. On the other hand, the present study aims to present real-world data. Second, the determination of permanent hypothyroidism is unclear since different criteria are used widely. Reactive hypothyroidism typically follows the first phase of destructive thyrotoxicosis in the natural progression of SAT. After the inflammation has subsided, new thyroid follicular cells are generated, resulting in full thyroid function restoration in the vast majority of patients [7]. Although restoration processes may last from 2 to 8 weeks, the exact time of proper evaluation of thyroid function is not elucidated. To verify long-term hormone replacement necessity, discontinuation, and reevaluation are necessary, particularly in patients with L-thyroxine substitution early in the course of the disease. This phenomenon leads to overdiagnosis of hypothyroidism, and thus the different incidences of hypothyroidism reported in the literature may not be comparable. In all patients with thyroxine administration enrolled in our study, a trial to discontinue treatment was attempted. Permanent hypothyroidism, defined as TSH values > 4.5 IU/mL, was disclosed in 32% of patients.

Third, published data about the exact dose of GCs as well as the tapering period (including our study) are frequently incomplete. Well-designed cohort studies should be conducted to clarify the optimal treatment protocol to minimize both the recurrence rate and hypothyroidism. Moreover, as the risk of recurrence may be human leukocyte antigen system (HLA)-dependent [10], interpretation of the results from various regions, even in the same country, may be biased. Although patients’ recruitment in the present study exhibits an excellent geographical distribution in a relatively small country, the HLA background is missing, and the unknown influence of HLA on treatment outcomes might be crucial. In addition, differential diagnosis of SAT is still challenging [40,41,42,43,44], particularly in the era of COVID disease and vaccination [36]. Considering that a significant proportion of patients use over-the-counter anti-inflammatory medications for symptom relief, the concern is to avoid false negative and, more importantly, false positive SAT diagnoses.

Lastly, missing information on prior clinicopathological features of patients and treatment details was available from the retrospective chart review as some patients presented to endocrine clinics following SAT diagnosis. This prevented further stratification based on the baseline hormonal profile, and the true prevalence of prior thyroid pathology cannot be accurately elucidated from this study as it requires a prospective design. However, given the rarity and significance of SAT, such data are rare and difficult to obtain. To the best of our knowledge, this study represents one of the first and largest studies to examine the differences between the well-known entity of SAT and its newly described form, the one related to recent COVID-19 infections, and the first to assess the efficacy of different treatment strategies in this uncommon patient population in a real-world case series study.

## 5. Conclusions

CRP may be used as a specific index for therapy discontinuation, according to our data originating from a large cohort of subjects of Caucasian origin. Our study confirmed previous reports in terms of the superiority of NSAIDs for the elimination of recurrence rates in SAT treatment. Both treatment strategies failed to prevent the onset of delayed hypothyroidism. A strong predictor of the onset of hypothyroidism was the existence of Hashimoto’s thyroiditis. Finally, the clinical features of SAT caused by COVID-19 infections were comparable to those of typical SAT disease. Extensive studies are needed to elucidate the association between clinical and laboratory characteristics and SAT recurrence. No definite treatment protocol is yet available for SAT, and treatment regimens followed vary substantially among various published articles. It is recommended to carry out more clinical studies, particularly with the combination of GCs and NSAIDs, to observe the prognosis and long-term effects of SAT patients.

## Figures and Tables

**Table 1:** (**A**) Clinical descriptive characteristics of this study population; (**B**) Laboratory characteristics of this study population.

(A)														
	Age (Year)	BMI (kg/m^2^)	Gender M/F, %	COVID-19	Recurrence	Time to Treat	Symptoms Relief	Treatment Duration	Symptoms Relief	Follow Up (Months)				
*n*, (%)			68/158 (30/70)	34−15.04	32−14.16					12				
Median	46	25.8				15	3	35	30	12				
Mean	48.01	25.98				20.39	5.155	45.27	39.27	16.77				
SD	11.31	4.642				19.65	6.465	37.05	39.99	11.82				
SEM	0.754	0.345				1.418	0.43	2.557	7.301	0.806				
Range	16–86	17–55				3–120	1–60	5–190	3–210	1–38				
Lower 95% CI of mean						17.59	4.33	40.23	24.33	15.19				
Upper 95% CI of mean						23.18	6.024	50.31	54.2	18.36				
(**B**)														
	**ESR**		**CRP**		**TSH**		**FT4**		**Tg**		**Anti-TPO**		**Anti-Tg**	
	**B**	**W**	**B**	**W**	**B**	**W**	**B**	**W**	**B**	**W**	**B**	**W**	**B**	**W**
Median	58.5	12	7.75	0.7	0.05	2.9	1.5	1.05	87	25	22	17	2	3
Mean	59.74	13.78	16.53	1.49	0.248	4.432	1.711	1.027	154.3	31.01	29.44	27.47	12.03	12.47
SD	25.32	8.93	24.76	1.871	0.503	5.241	0.649	0.224	206.1	28.67	31.34	30.9	27.4	26.06
SEM	1.756	0.6813	1.816	0.142	0.042	0.457	0.056	0.019	22.22	3.451	5.018	5.013	4.229	4.228
Range	4–133	2–76	0.1–129	0.0–9.3	0.004–3.8	0.1–38	0.45–6.02	0.5–1.89	1.2–1232	3–218	3–144	3–235	0.5–123	0.1–122
Lower 95% CI of mean	56.28	12.44	12.95	1.214	0.164	3.526	1.6	0.988	110.1	24.13	19.28	17.32	3.488	3.899
Upper 95% CI of mean	63.2	15.13	20.11	1.776	0.332	5.338	1.822	1.066	198.5	37.9	39.59	37.63	20.57	21.03

Footnote: Abbreviations: BMI, Body mass index; M/F, Male to Female; COVID-19, coronavirus disease 2019; Symptoms Relief, from the onset of SAT symptoms. Time is expressed in days unless otherwise indicated; ESR, erythrocyte sedimentation rate; CRP, C-reactive protein; TSH, Thyroid-stimulating hormone; FT4, Free thyroxin; Tg, Thyroglobulin; Anti-Tpo, Anti-thyroid peroxidase antibodies; Anti-Tg, Antithyroglobulin antibody; B, Baseline; W, Withdrawal.

**Table 2:** Characteristics of patients according to COVID-19 infection and recurrences.

Variable	COVID-19 *n* = 34	Non-COVID-19 *n* = 192	*p* Value	Recurrence *n* = 32	No-Recurrence *n* = 194	*p* Value
Age (y), mean (SD)	47.53 ± 10.42	48.32 ± 11.70	0.731	45.81 ± 10.27	53.53 ± 41.72	0.217
BMI (kg/m^2^), mean (SD)	27.58 ± 4.05	25.69 ± 5.44	**0.026**	25.33 ± 4.04	26.12 ± 4.75	0.562
Gender (M/F)	7/27	61/131	0.227 ^b^	10/22	58/136	0.838 ^b^
ESR (mm/h), mean (SEM)	62.45 ± 4.22	60.32 ± 2.05	0.682 ^a^	65.30 ± 4.60	58.80 ± 1.89	0.194 ^a^
ESR withdrawal (mm/h), mean (SEM)	13.32 ± 6.08	13.88 ± 9.40	0.811	15.60 ± 1.54	13.48 ± 0.75	0.148
CRP (mg/L), mean (SD)	17.72 ± 22.36	17.11 ± 26.62	0.690	21.18 ± 31.54	28.80 ± 165.6	0.437
CRP withdrawal (mg/L), mean (SD)	1.674 ± 1.87	1.468 ± 1.87	0.261	2.532 ± 2.63	1.336 ± 1.68	**0.029**
Time to treat (days), mean (SD)	23.45 ± 27.64	20.13 ± 19.94	0.752	17.40 ± 11.62	20.94 ± 20.78	0.877
Therapy (days), mean (SD)	46.64 ± 32.54	48.01 ± 40.74	0.564	41.69 ± 26.00	45.91 ± 38.73	0.726
Time to relief (days), mean (SD)	3.56 ± 1.94	5.46 ± 6.93	**0.044**	3.90 ± 1.78	5.387 ± 6.92	0.912
Recurrence, *n* (%)	5 (14.7)	27(14.1)	0.541 ^b^			
Time to recurrence, mean (SD)	30.67 ± 26.95	42.44 ± 41.49	0.663	39.27 ± 39.99	-	
Hypothyroidism development, N (%)	9 (26.5)	60(31.2)	0.810 ^b^	6	63	0.148 ^b^
Cortisone, *n*	28	128	0.073 ^b^	30	126	**<0.001** ^b^
Initial dose (mg), mean (SD)	27.29 ± 8.22	22.85 ± 8.83	**0.015** ^a^	26.27 ± 9.89	23.00 ± 8.55	0.084
Methylprednisolone, *n*	23	86	* 0.117 ^b^	26	83	* **0.027** ^b^
Initial dose (mg), mean (SD)	28.52 ± 7.56	26.07 ± 7.62	0.177 ^a^	28.31 ± 8.23	26.05 ± 7.41	0.374
Prednisolone, *n*	5	35	* 0.348 ^b^	4	36	* 0.105 ^b^
Initial dose (mg), mean (SD)	21.50 ± 9.63	21.49 ± 8.62	0.936	13.00 ± 10.52	22.44 ± 8.01	0.087
Dexamethasone, *n*	-	7		-	7	
Initial dose (mg), mean (SD)	-	16		-	16	
Ibuprofen, *n*	4	38	0.343 ^b^	2	40	0.053 ^b^
Initial dose (mg), mean (SD)	1200 ± 0.00	1058 ± 310	**0.016** ^a^	1200 ± 0.00	1067 ± 302.8	0.716
Combined treatment, *n*	-	15		-	15	
Initial dose (mg)	-	10.93 ± 3.53		-	10.93 ± 3.53	
None, *n*	2	11	0.989 ^b^	-	13	

Footnote: Comparisons between the two groups were made using the Student unpaired *t*-test **^a^** and the Mann–Whitney U test or Fisher’s exact test **^b^**, as appropriate. * Concerning patients under cortisone therapy. Abbreviations: BMI, Body mass index; M/F, Male to Female; ESR, erythrocyte sedimentation rate; CRP, C-reactive protein; COVID-19, coronavirus disease 2019; Time to relief, from the onset of SAT symptoms; Initial doses are equivalent to steroidal doses of methylprednisolone. Bold values are considered significant at α level < 0.05.

## Data Availability

Some or all datasets generated during and/or analyzed during the current study are not publicly available but are available from the corresponding author upon reasonable request.
